# Supplementary material for: A rapid, accurate, scalable, and portable testing system for COVID-19 diagnosis
Source: Nat Commun. 2021 May 18;12:2905. doi: 10.1038/s41467-021-23185-x (PMC8131735; doi:10.1038/s41467-021-23185-x)

SUPPLEMENTARY INFORMATION

A Rapid, Accurate, Scalable and Portable Testing System for COVID-19 Diagnosis

Guanhua Xun[1,#], Stephan Lane[2,#], Vassily Petrov[2], Brandon Pepa[3], and Huimin Zhao[1,2,4,*]
[1]Department of Bioengineering,
[2]Carl R. Woese Institute for Genomic Biology,
[3]Department of Mechanical Engineering,
[4]Departments of Chemical and Biomolecular Engineering, Chemistry, and Biochemistry,

University of Illinois at Urbana-Champaign, Urbana, IL 61801
[#]These authors contributed equally
*Correspondence to: zhao5@illinois.edu.

Table of content

1. Supplementary figures page 2-13
2. Supplementary table page 14
3. User guide for the SPOT testing system page 15-17

SUPPLEMENTARY FIGURES

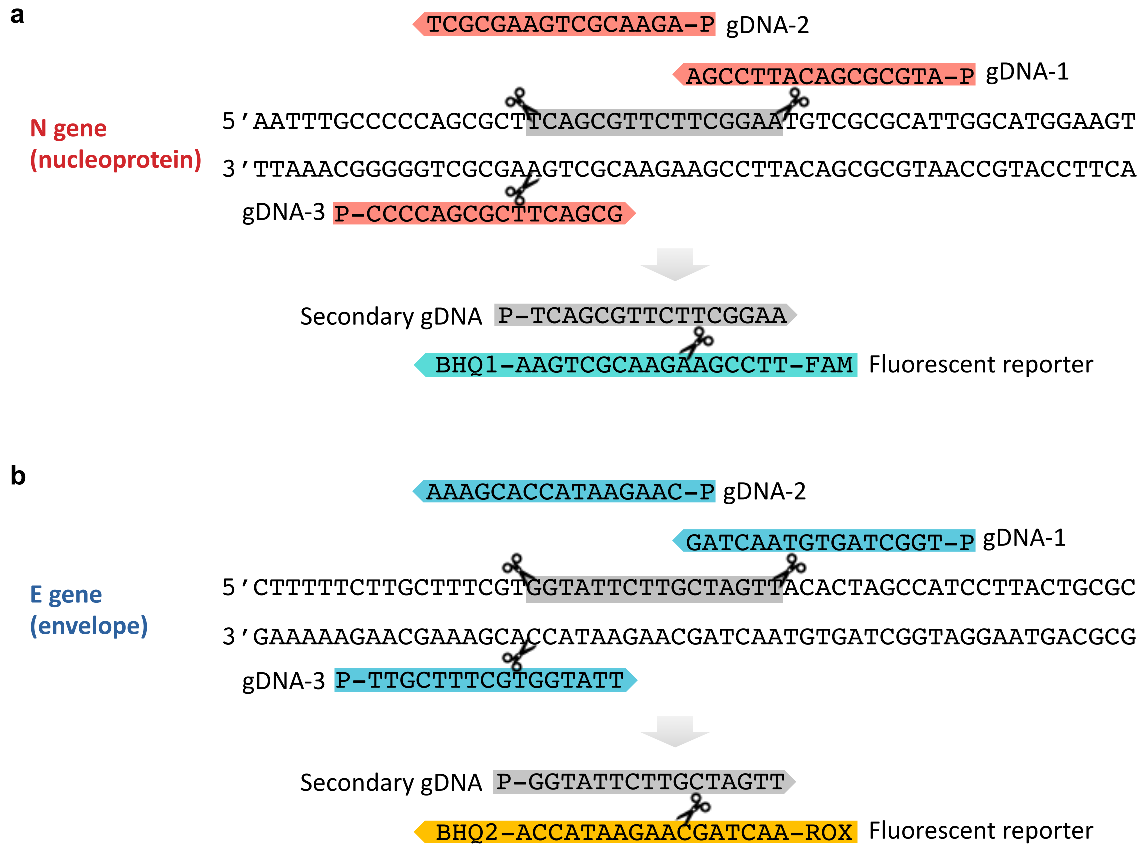

Supplementary Figure 1. Mechanism of SPOT reaction. (a) The gDNA-1 and gDNA-2 for N gene generate the secondary gDNA by cleaving the sense strand of N gene amplicon. The gDNA-3 helps to release the secondary gDNA from the primary cleavage. The secondary gDNA targets to the complementary fluorescent reporter to mediate the cleavage on reporter by *Pf*Ago. **(b)** The gDNA-1 and gDNA-2 for E gene generate the secondary gDNA by cleaving the sense strand of E gene amplicon. The gDNA-3 helps to release the secondary gDNA from the primary cleavage. The secondary gDNA targets to the complementary fluorescent reporter to mediate the cleavage on reporter by *Pf*Ago.

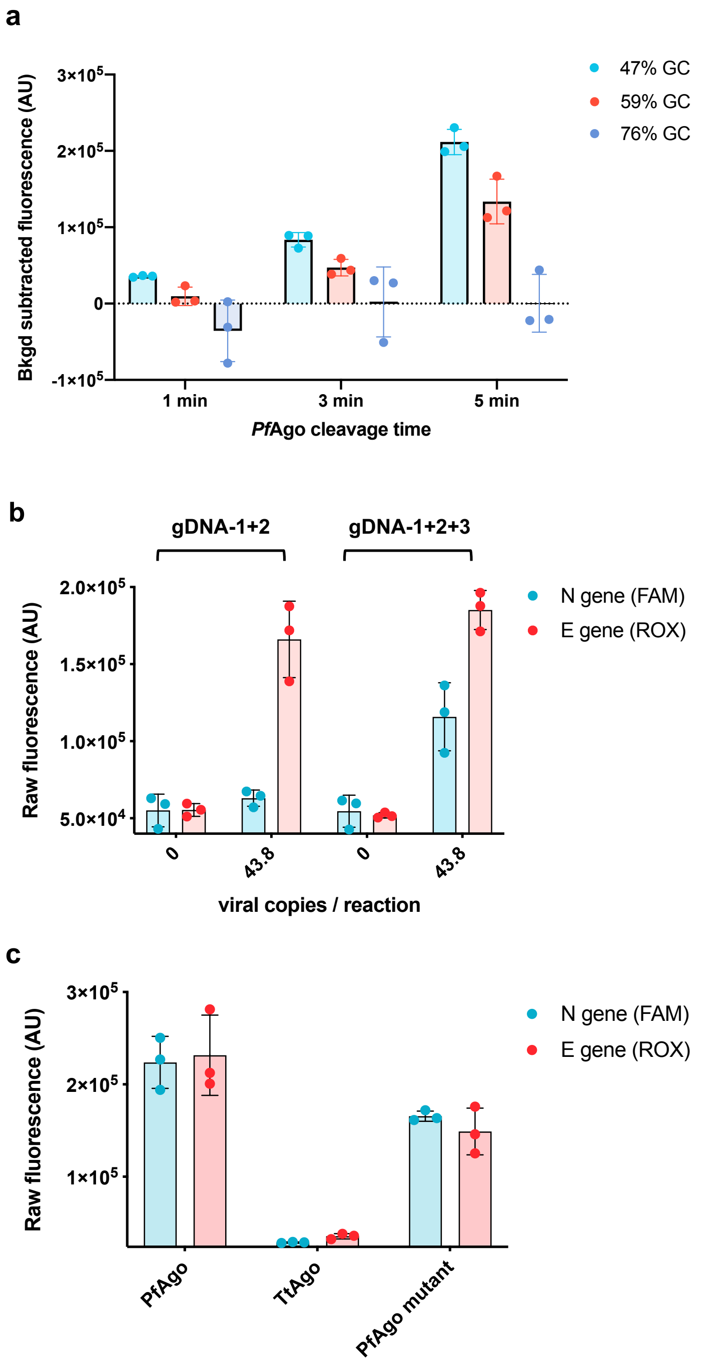

Supplementary Figure 2. Cleavage efficiency and optimization of SPOT assay. (a) Optimization of GC content of the fluorescent reporters. The GC contents of three fluorescent reporters are 76%, 59% and 47%, respectively. Fluorescent signals were collected after 1 min, 3 min, and 5 min *Pf*Ago cleavage with the same amount of LAMP products input (n = 3 biologically independent experiments). The net fluorescence value increases were calculated by subtracting the initial background fluorescence value in order to reduce the difference in fluorescence intensity between different reporters. **(b)** Comparison of cleavage efficiency between combinations of gDNAs (n = 3 biologically independent experiments). **(c)** Optimization of the fluorescence-based nucleic acid detection reaction. Three candidate enzymes were added at the same amount. *Tt*Ago mediated reaction was incubated at 80°C without manganese ion (Mn^{2+}). *Pf*Ago mediated reaction was performed at 95°C with manganese ion (Mn^{2+}). Optimized *Pf*Ago mutant, which shows cleavage activity in the presence of magnesium ion (Mg^{2+}), was added to the reaction at 95°C (n = 3 biologically independent experiments). Error bars represents the mean with standard deviations. Source data are provided as a Source Data file.

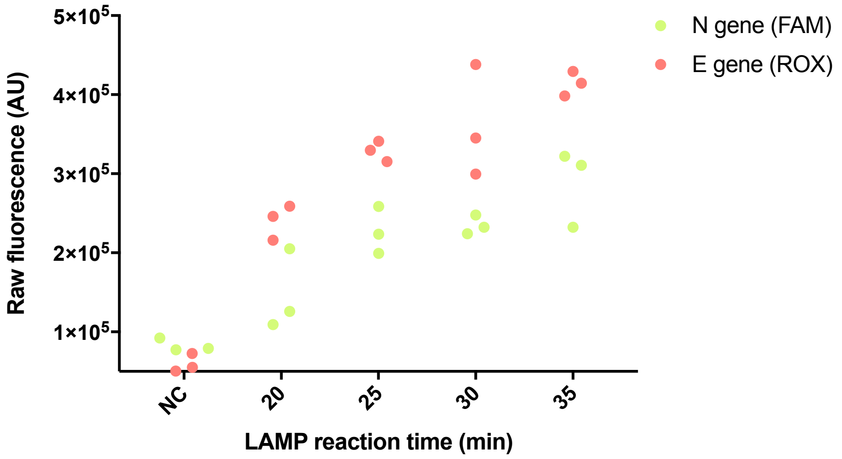

Supplementary Figure 3. Optimization of the LAMP reaction time. Saliva samples spiked with 43.8 copies of gamma-irradiated SARS-CoV-2 virus were added into the RT-LAMP reaction and incubated at 63°C for different lengths of time (n = 3 biologically independent experiments). Source data are provided as a Source Data file.

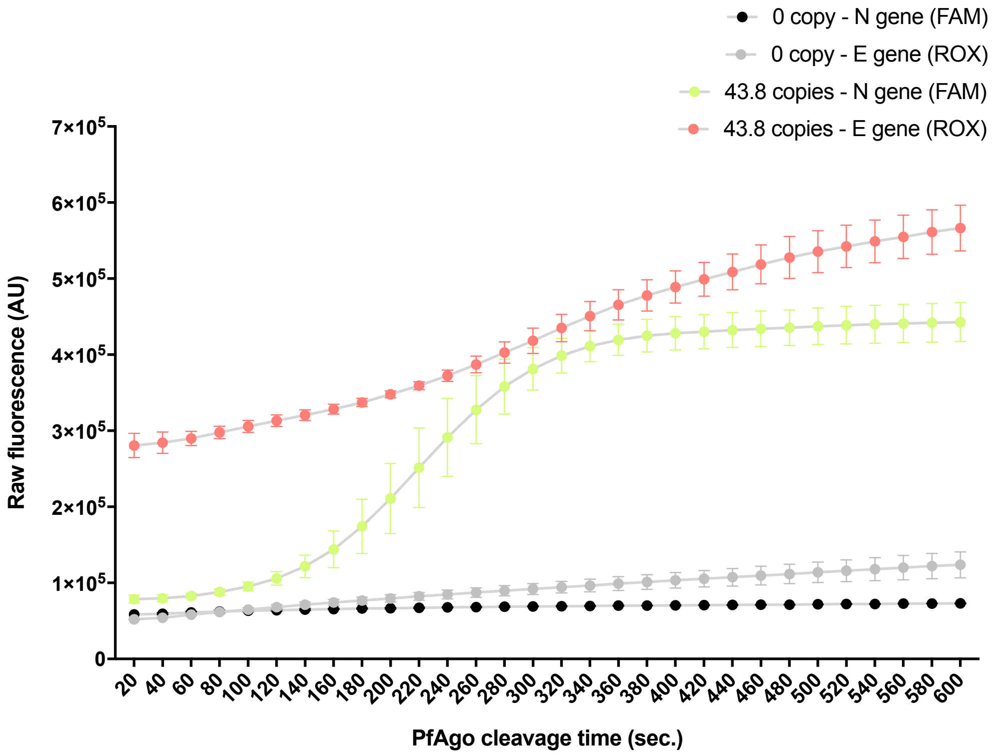

Supplementary Figure 4. Optimization of *Pf*Ago cleavage reaction time. A saliva sample spiked with 43.8 copies of gamma-irradiated SARS-CoV-2 virus was added into the RT-LAMP reaction mixture and incubated at 63°C for 30 min. *Pf*Ago was then added into the RT-LAMP reaction product and the fluorescence signal was determined by a real-time PCR machine with the following program: 20 sec. at 95°C, 5 sec. at 25°C for capturing the fluorescent signal, a total of 30 cycles (n = 3 biologically independent experiments). Error bars represents the mean with standard deviations. Source data are provided as a Source Data file.

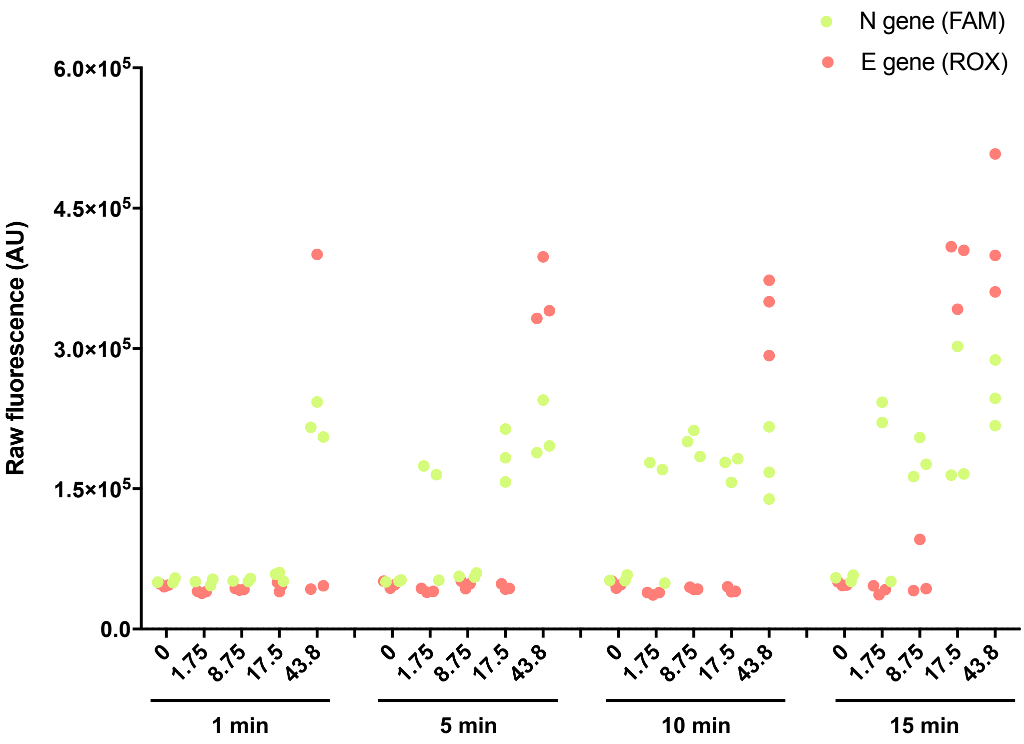

Supplementary Figure 5. Optimization of saliva sample pre-treatment time. Saliva samples spiked with 0, 1.75, 8.75, 17.5, and 43.8 copies of gamma-irradiated SARS-CoV-2 virus were mixed with QuickExtract DNA Extraction Solution at a 1:1 ratio and heated at 95°C for 1 min, 5 min, 10 min and 15 min, respectively (n = 3 biologically independent experiments). Source data are provided as a Source Data file.

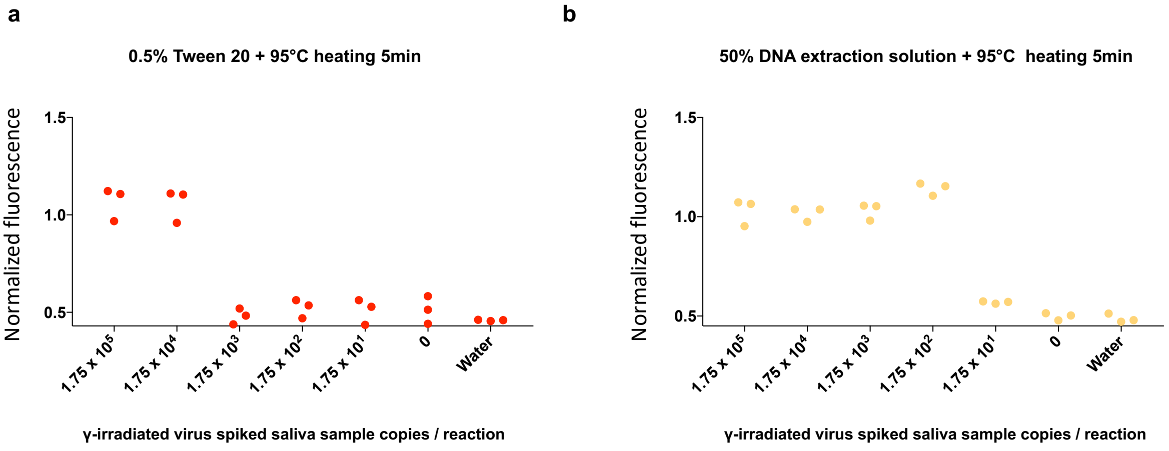

Supplementary Figure 6. Additives for enhancing viral genome release. Tween 20 and DNA extraction solution were explored for their ability to enhance SARS-CoV-2 detection. Either 0.5% Tween 20 (panel **a**) or 50% DNA extraction solution (Lucigen, Inc.) (panel **b**) was added to a saliva sample spiked with gamma irradiated SARS-CoV-2 virus and heated at 95°C for 5 min (n = 3 biologically independent experiments). Source data are provided as a Source Data file.

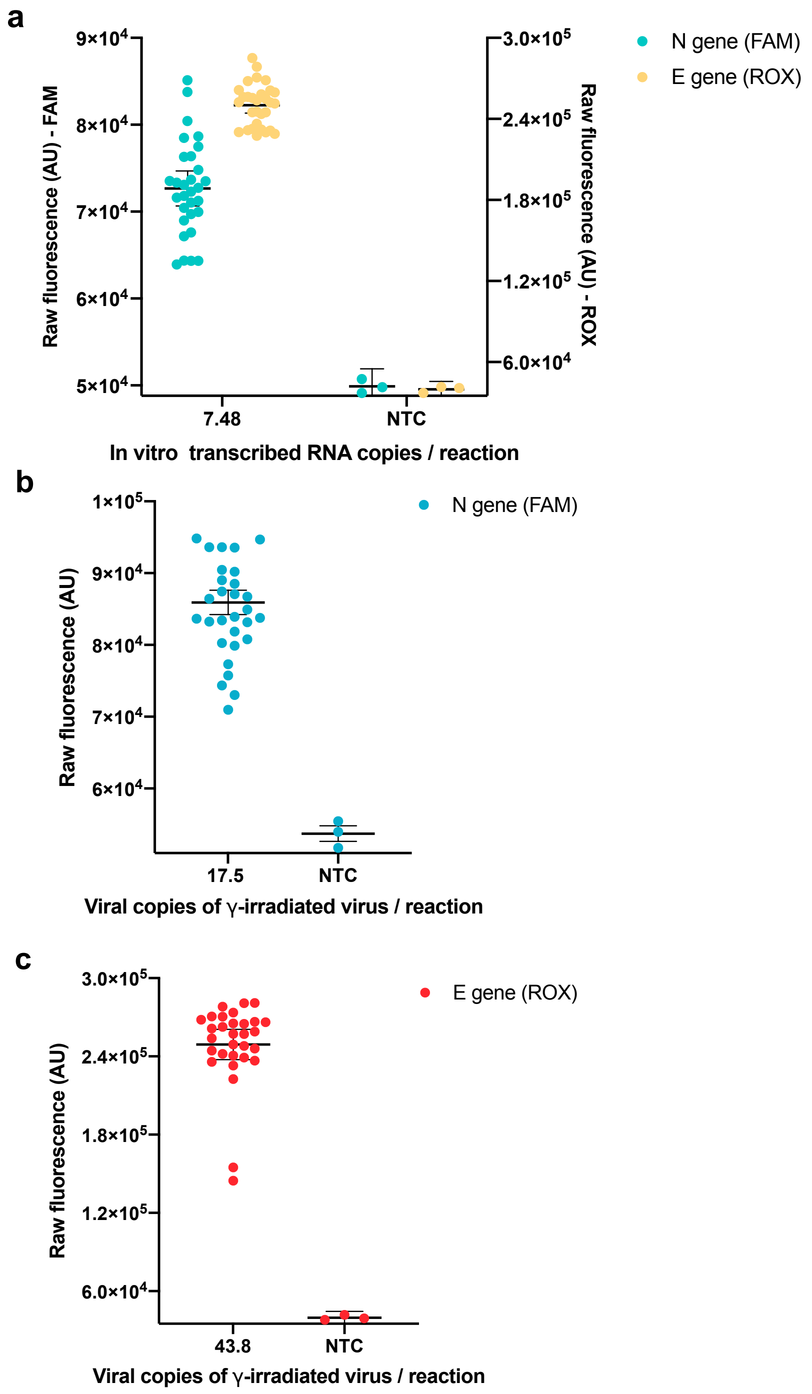

Supplementary Figure 7. LoD reliability and reproducibility of SPOT assay. (a) 30 replicates of in vitro transcribed RNA spiked saliva samples with the LoD concentration (7.48 copies/rxn) were detected by SPOT assay (n = 30 biologically independent experiments). **(b)** 30 replicates of γ-irradiated virus spiked saliva sample with the N gene LoD concentration (17.5 copies/rxn) were detected by SPOT assay (n = 30 biologically independent experiments). **(c)** 30 replicates of γ-irradiated virus spiked saliva sample with the E gene LoD concentration (43.8 copies/rxn) were detected by SPOT assay (n = 30 biologically independent experiments). All samples were incubated on a thermocycler and measured on a qPCR machine (QuantStudio 3 RT-PCR system). Error bar represents the mean with 95% CI. Source data are provided as a Source Data file.

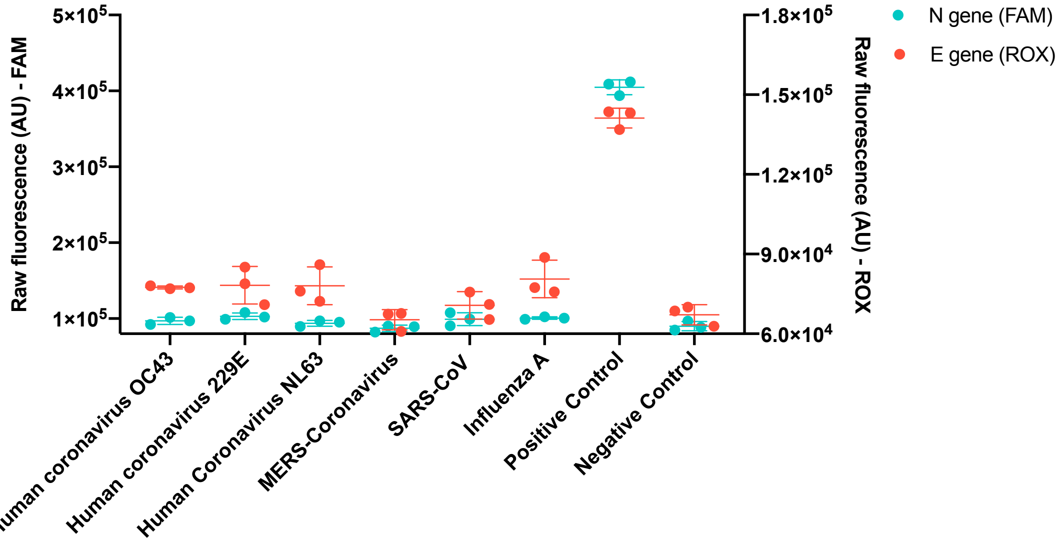

Supplementary Figure 8. Specificity of SPOT assay on SARS-CoV-2 among other respiratory viruses. Commercially available saliva (Innovative Research) were spiked with human coronaviruses (OC43, 229E and NL63) genomic RNA, SARS and MERS viruses (γ-irradiated), and influenza A genomic RNA, and diluted 1:1 with QuickExtract DNA Extraction Solution. Samples were heat treated at 95°C for 5 min. Virus-spiked saliva samples, a positive control (γ-irradiated SARS-CoV-2) and a negative control were incubated on thermocycler, and measured on a qPCR machine (QuantStudio 3 RT-PCR system) (n = 3 biologically independent experiments). Error bars represents the mean with standard deviations. Source data are provided as a Source Data file.

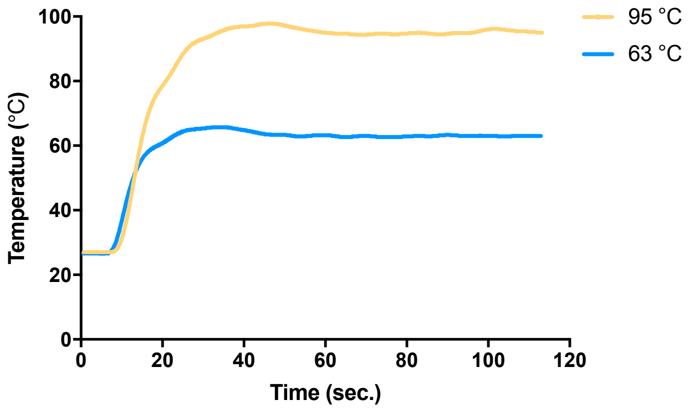

Supplementary Figure 9. Temperature profile of the thermal module in the SPOT device. The SPOT device heated the capillary to 63°C and 95°C in 25 seconds and 30 seconds, respectively. Temperature data was collected from a temperature sensor placed inside a capillary. Source data are provided as a Source Data file.

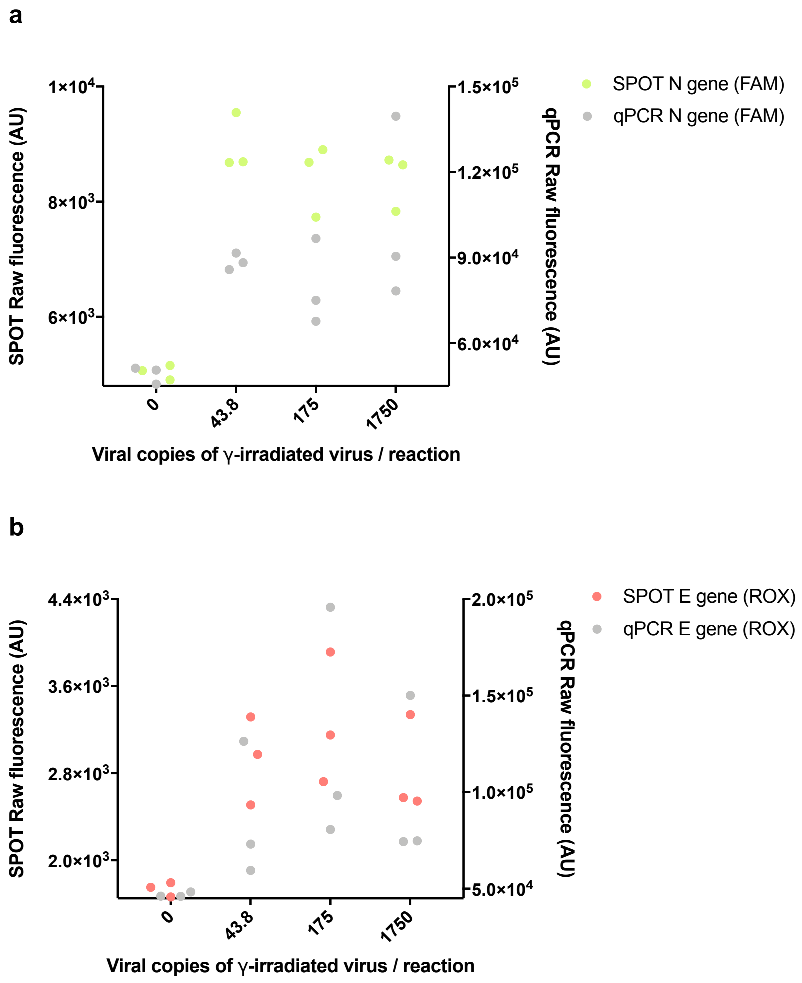

Supplementary Figure 10. Comparison of sensitivity of fluorescence quantification between SPOT device and qPCR machine. To analyze SPOT assay results using SPOT device fluorescence quantification., the SPOT assays were performed on a thermocycler and then transferred to a capillary for fluorescent quantification using the SPOT device (green dots shown in panel **a** represent the N gene associated FAM fluorescence, red dots shown in panel **b** represent the E gene associated ROX fluorescence). As a comparison, those samples were also measured on a qPCR machine (QuantStudio 3 RT-PCR system), marked in grey dots in both "a" and "b". SPOT assays were performed using gamma irradiated virus spiked saliva with initial copies of viral particles at 0, 43.8, 175, and 1750 viral copies per reaction (n = 3 biologically independent experiments). Source data are provided as a Source Data file.

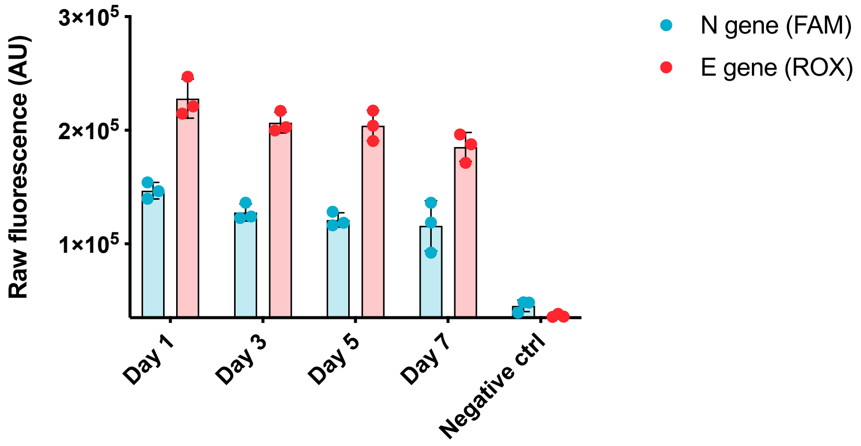

Supplementary Figure 11. Stability of prefabricated reaction capillary. Reaction capillaries were prepared using LAMP and *Pf*Ago chambers and stored at -20°C. The capillaries were taken out to measure the activity over time (n = 3 biologically independent experiments). Error bars represents the mean with standard deviations. Source data are provided as a Source Data file.

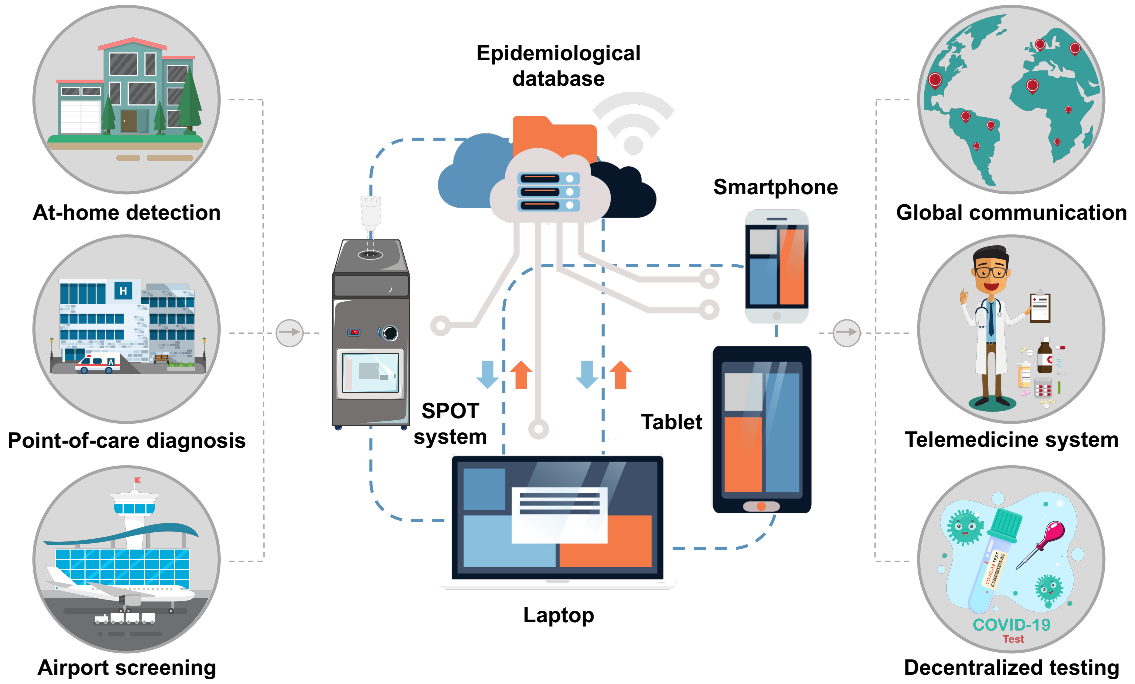

Supplementary Figure 12. Perspective of the SPOT device. By connecting the SPOT system with networked devices, such as laptops, tablets, and smartphones, the advanced capabilities such as connectivity, databasing, and data sharing enable better global pandemic controlling, platform of telemedicine, and decentralized testing.

Supplementary Table 1. Clinical evaluation of the SPOT testing system.

ID	SPOT readouts		qRT-PCR interpretation	Ct value		
	N gene	E gene		S gene	N gene	ORF gene
1	POS	POS	POS	27.6	26.6	27.1
2	POS	POS	POS	26.0	24.8	25.0
3	POS	POS	POS	21.7	18.1	20.8
4	POS	POS	POS	19.4	17.5	19.0
5	POS	POS	POS	27.5	26.0	27.2
6	POS	POS	POS	26.6	22.8	25.9
7	POS	POS	POS	29.5	27.2	29.7
8	NEG	NEG	POS	32.7	32.6	33.1
9	NEG	NEG	NEG			
10	POS	POS	POS	31.6	28.7	30.1
11	NEG	NEG	NEG			
12	NEG	NEG	NEG			
13	NEG	NEG	NEG			
14	NEG	NEG	NEG			
15	NEG	NEG	NEG			
16	NEG	NEG	NEG			
17	POS	POS	POS	29.1	26.8	28.1
18	POS	POS	POS	33.3	30.2	31.3
19	NEG	NEG	NEG			
20	NEG	NEG	NEG			
21	NEG	NEG	NEG			
22	NEG	NEG	POS	36.5	35.6	36.6
23	POS	POS	POS	29.5	26.6	27.8
24	NEG	NEG	NEG			
25	NEG	NEG	NEG			
26	NEG	NEG	NEG			
27	POS	POS	POS	27.9	27.6	27.6
28	NEG	NEG	NEG			
29	NEG	NEG	NEG			
31	NEG	NEG	NEG			
32	POS	POS	POS	35.1	32.0	neg
33	POS	POS	POS	26.5	24.9	26.0
34	POS	POS	POS	23.7	21.9	23.4
36	POS	POS	POS	32.1	29.9	31.6
37	POS	POS	POS	29.3	28.1	28.8
38	POS	POS	POS	27.3	25.9	26.5
39	POS	POS	POS	24.6	22.9	24.8
40	NEG	NEG	NEG			
41	NEG	NEG	NEG			
42	POS	POS	POS	25.6	24.5	25.9
43	POS	POS	POS	21.4	20.8	21.4
44	NEG	NEG	NEG			
45	POS	POS	POS	30.5	29.3	30.8
46	NEG	NEG	NEG			
47	POS	POS	POS	32.3	31.3	33.6
48	NEG	NEG	NEG			
49	NEG	NEG	NEG			
50	POS	POS	POS	22.2	21.2	21.9
51	NEG	NEG	NEG			
52	NEG	POS	NEG			
53	NEG	NEG	POS			
54	NEG	NEG	NEG			
55	NEG	NEG	NEG			
56	NEG	NEG	NEG			
57	NEG	NEG	NEG			
58	POS	POS	POS	25.2	23.4	25.1
59	POS	POS	POS	22.1	21.2	21.9
60	NEG	NEG	NEG			
61	NEG	NEG	NEG			
62	NEG	NEG	NEG			
63	NEG	NEG	NEG			
64	NEG	NEG	NEG			
65	NEG	NEG	NEG			
66	NEG	NEG	NEG			
67	NEG	NEG	NEG			
68	NEG	NEG	NEG			
69	NEG	NEG	NEG			
70	NEG	NEG	NEG			
71	NEG	NEG	NEG			
71	NEG	NEG	NEG			
73	NEG	NEG	NEG			
74	NEG	NEG	NEG			
75	NEG	NEG	NEG			
76	NEG	NEG	NEG			
77	NEG	NEG	NEG			
78	NEG	NEG	NEG			
79	NEG	NEG	NEG			
80	NEG	NEG	NEG			
81	NEG	NEG	NEG			
82	POS	POS	POS	34.5	30.1	35.2
83	NEG	NEG	NEG			
84	NEG	NEG	NEG			
85	NEG	NEG	NEG			
86	NEG	NEG	NEG			
87	NEG	NEG	NEG			
88	NEG	NEG	NEG			
89	NEG	NEG	NEG			
90	NEG	NEG	NEG			
91	NEG	NEG	NEG			
92	NEG	NEG	NEG			
93	NEG	NEG	NEG			
94	NEG	NEG	NEG			
95	NEG	NEG	NEG			
96	NEG	NEG	NEG			
97	NEG	NEG	NEG			
98	NEG	NEG	NEG			
99	NEG	NEG	NEG			
100	NEG	NEG	NEG			
101	NEG	NEG	NEG			
102	NEG	NEG	NEG			
103	NEG	NEG	NEG			
104	NEG	NEG	NEG			
105	POS	POS	POS	29.9	27.5	29.7
106	NEG	NEG	NEG			

SUPPLEMENTARY TEXT

User guide for the SPOT device

1. Turn on the device by pressing the “power” button. The system will initialize with following display on the screen, then the LED indicator will turn blue.

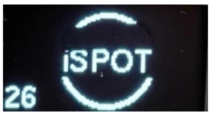

2. Insert capillary 1 into the SPOT device and press the “reaction” button to initiate the pretreatment step. The screen will display “reaction 1” and the indicator will turn to green.

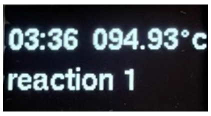

3. Following the 5-min pretreatment, remove capillary 1 and use the provided collection capillary to transfer a small volume from capillary 1 into capillary 2.

4. Insert capillary 2 into the SPOT device and press the “reaction” button again to initiate the detection reaction. The screen will display “reaction 2” and begin heating the capillary to 63°C.

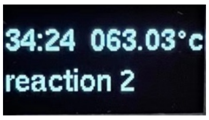

5. After 35 min, the fan will be turned on automatically to cool down the capillary. Following the 1-min cooldown period, the detection result will be shown on the screen.

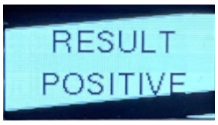

User guide for the SPOT dashboard system

Connect a computer to the SPOT device using a USB-C cable and run the SPOT dashboard. The SPOT dashboard system allows you to measure samples, modify device settings, and export data.

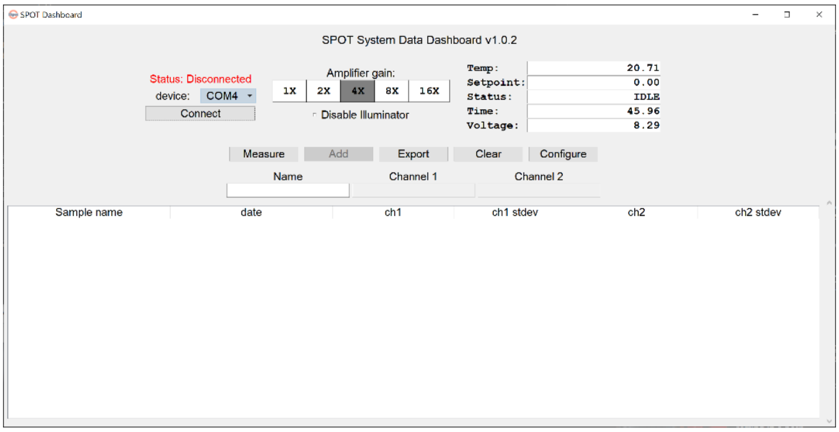

Power on the SPOT device and press the “Connect” button to start receiving information and data from the SPOT device.

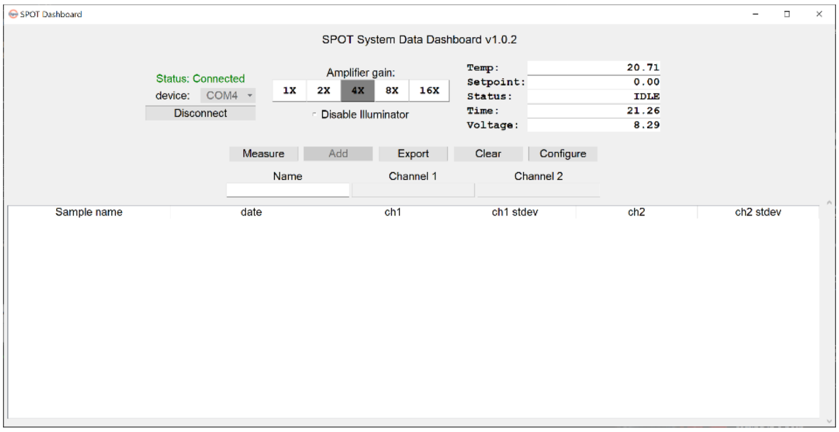

Press the “Configure” button to modify device settings. The incubation time and target temperature for two reaction steps can be modified.

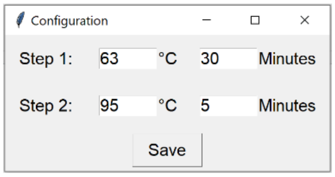

The data panel provides real-time information on the SPOT device’s current temperature, target temperature, reaction in progress, time connected to the SPOT dashboard, and a battery voltage. “REACTION 1” corresponds to the pretreatment step (95°C for 5 minutes by default), while “REACTION 2” corresponds to the detection reaction (63°C for 30 minutes followed by 95°C for 5 minutes, by default).

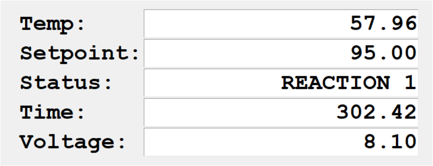

Use the 1x, 2x, 4x, 8x, or 16x buttons to adjust the amplifier gain as desired.

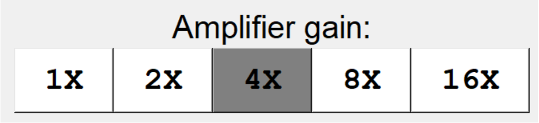

Insert a sample and press the "Measure" button to obtain a fluorescent measurement on channel 1 (FAM) and channel 2 (ROX). The mean of 16 consecutive fluorescent measurements and a standard deviation are saved for each sample.

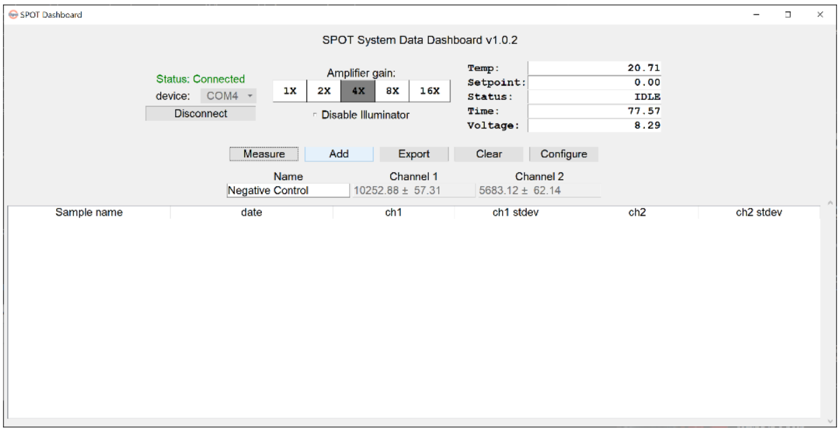

In the text box under "Name", provide a descriptive sample name then press the "Add" button to save the data point.

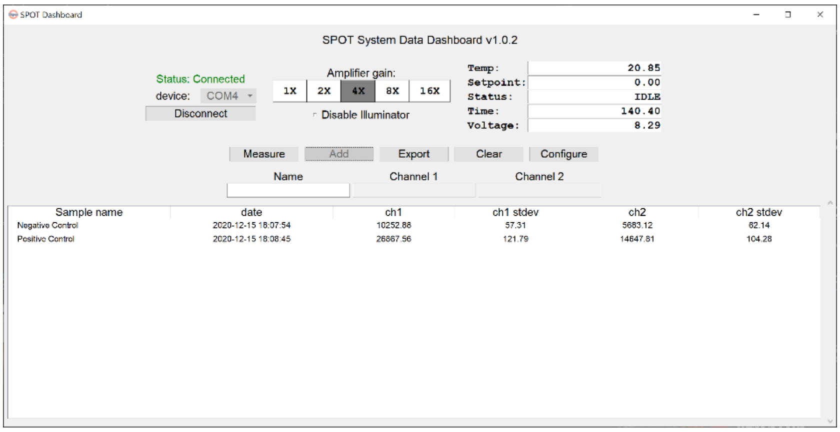

Press the "Export" button to save all recorded sample points to a comma-separated values (.csv) file.

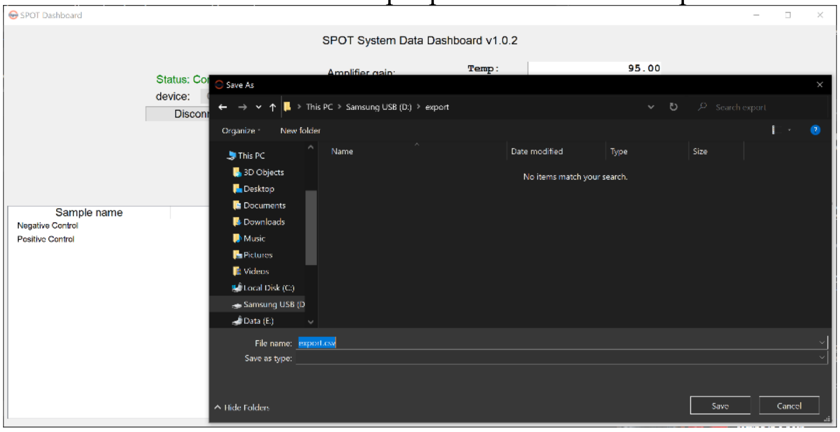

Supplement: Supplementary file 1 — Supplementary Information [file 41467_2021_23185_MOESM1_ESM.pdf]
