# Supplementary material for: A rapid, accurate, scalable, and portable testing system for COVID-19 diagnosis
Source: Nat Commun. 2021 May 18;12:2905. doi: 10.1038/s41467-021-23185-x (PMC8131735; doi:10.1038/s41467-021-23185-x)
Supplement: Supplementary file 2 — Supplementary Data 1 [file 41467_2021_23185_MOESM2_ESM.pdf]

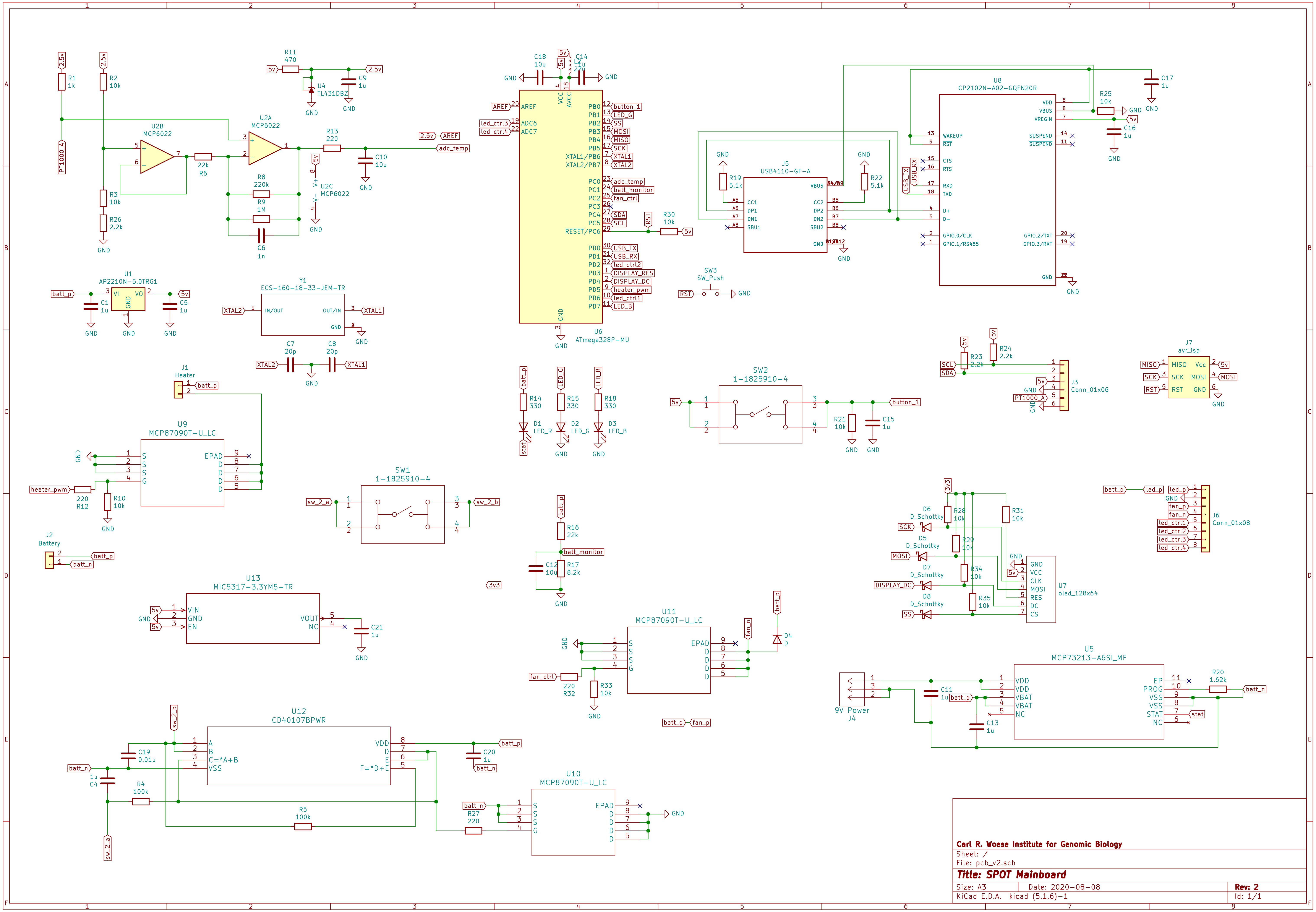

Carl R. Woese Institute for Genomic Biology

Sheet: /

File: pcb_v2.sch

Title: SPOT Mainboard

Size: A3 | Date: 2020-08-08 | Rev: 2

KiCad E.D.A. kicad (5.1.6)-1 | Id: 1/1

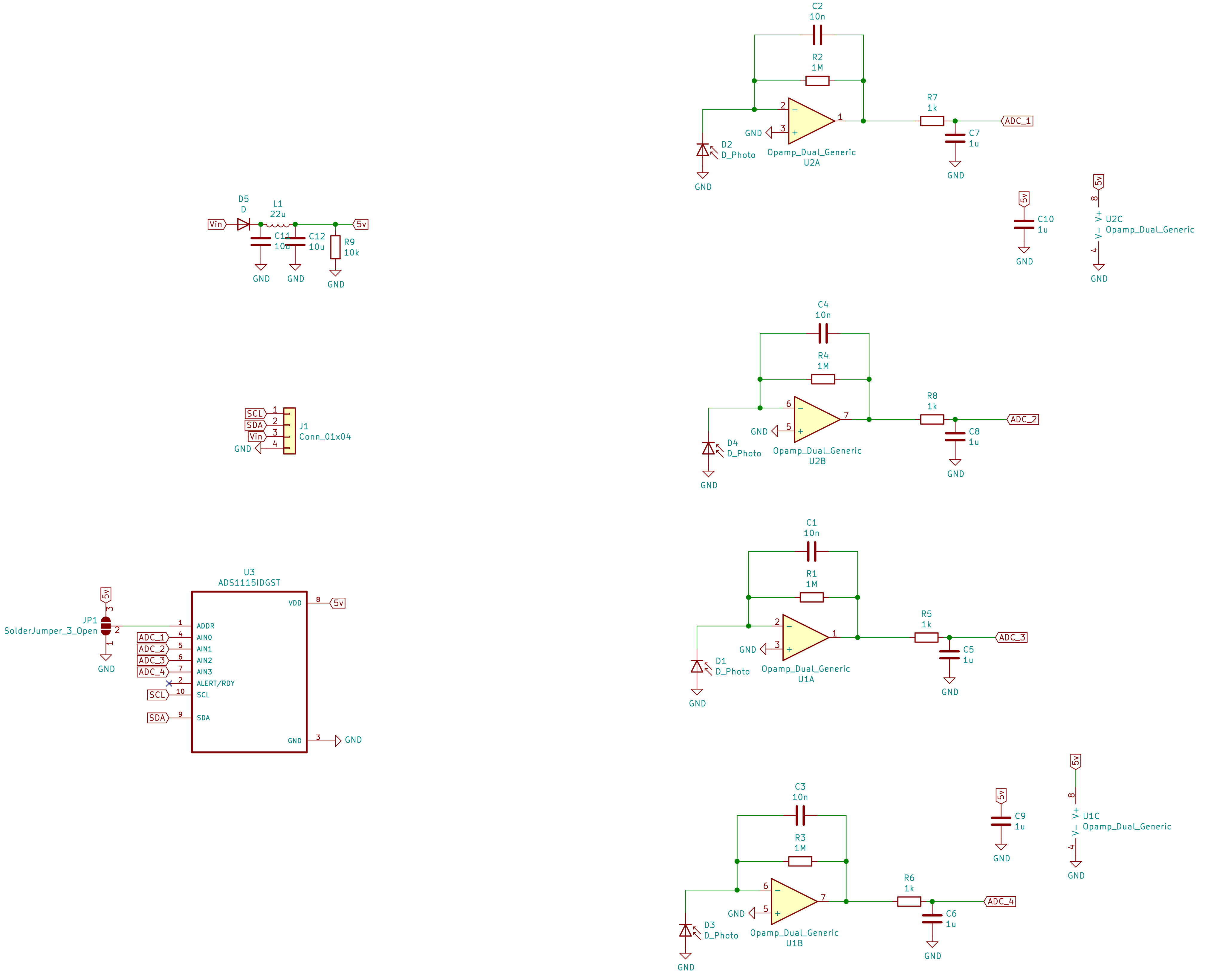

Carl R. Woese Institute for Genomic Biology

Sheet: /

File: pcb_photodiode_v2.sch

Title: SPOT Fluorescence Meter

Size: A3 | Date: 2020-08-08 | **Rev: 2**

KiCad E.D.A. eeschema (5.1.6)-1 | Id: 1/1

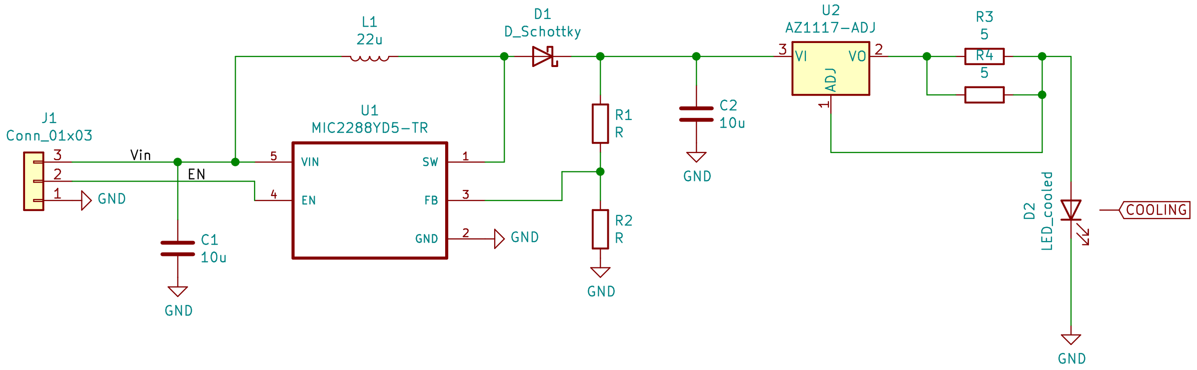

Carl R. Woese Institute for Genomic Biology

Sheet: /

File: uv_led_mount.sch

Title: SPOT UV Illuminator

Size: A3 Date: 2020-08-08 **Rev: 1**

KiCad E.D.A. eeschema (5.1.6)-1 Id: 1/1

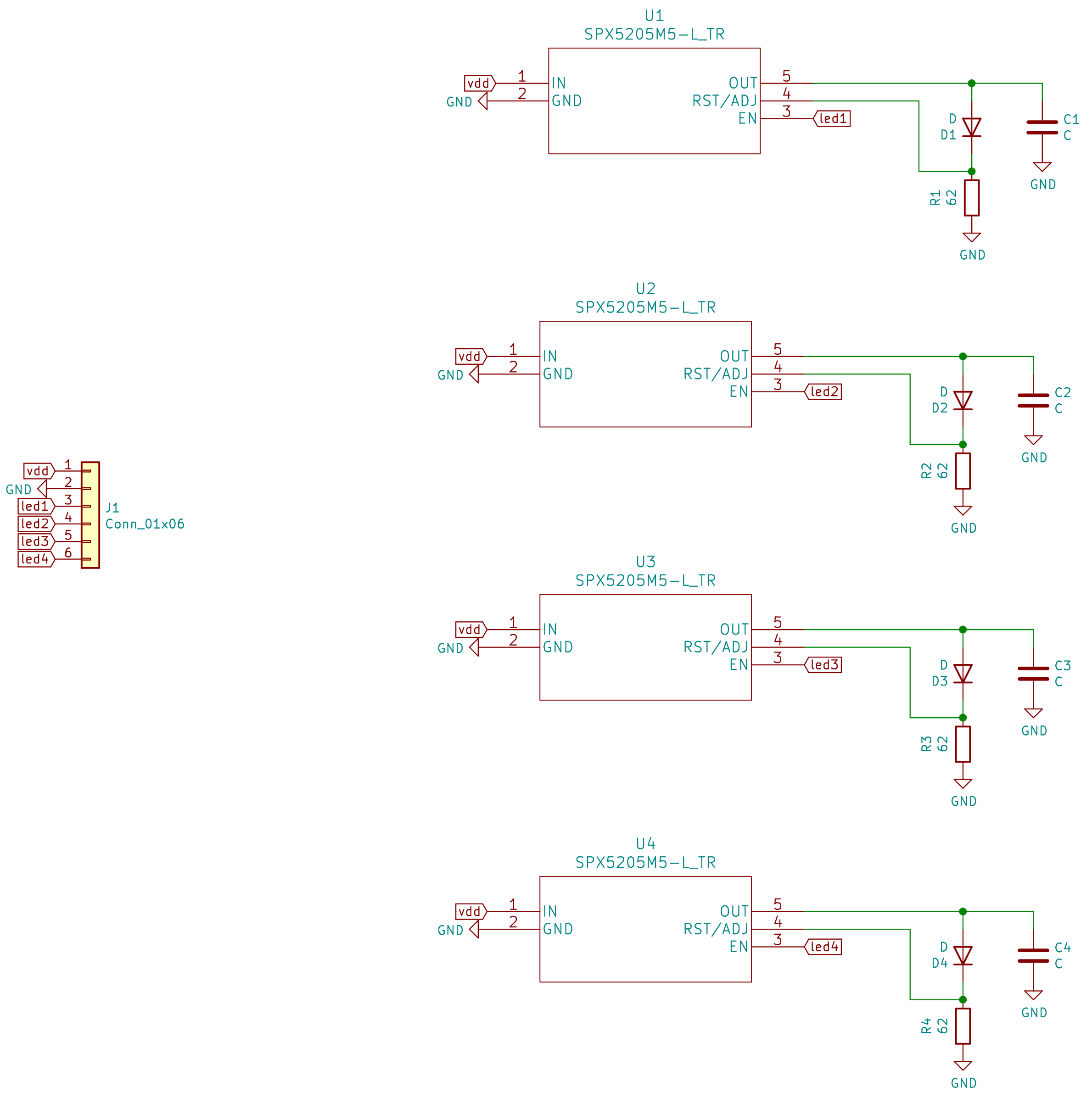

Carl R. Woese Institute for Genomic Biology

Sheet: /

File: pcb_led_v2.sch

Title: SPOT Visible Light Illuminator

Size: A3	Date: 2020-08-08	**Rev: 2**
KiCad E.D.A. kicad (5.1.6)-1		Id: 1/1
