# Supplementary material for: A rapid, accurate, scalable, and portable testing system for COVID-19 diagnosis
Source: Nat Commun. 2021 May 18;12:2905. doi: 10.1038/s41467-021-23185-x (PMC8131735; doi:10.1038/s41467-021-23185-x)
Supplement: Supplementary file 7 — Description of Additional Supplementary Files [file 41467_2021_23185_MOESM7_ESM.pdf]

Title: Supplementary Data 1.
Description: SPOT circuit schematics

The SPOT device is comprised of three independent circuit boards: **a**) The main circuit board houses a microcontroller which encodes the heating and timing parameters of the system. It is also responsible for reading temperature of the heater, regulating heat output, charging the battery, controlling the fan, displaying the user interface, and optionally communicating data to a PC via USB. **b**) The second circuit board holds the photodiodes which read and amplify the fluorescent output then convert it to a digital signal. This signal is then sent to the microcontroller on the main circuit board. One of two final circuit boards is included depending on the design and is used to excite the fluorophores in the system. **c**) A single high-power blue LED can be used to excite multiple fluorophores or four parallel standard LEDs with emission wavelengths near the chosen fluorophores' excitation maxima.

Title: Supplementary Data 2.
Description: SPOT bill of materials

The electronics and optical filters used in assembling the SPOT device total to $77.62 per device. The total cost of reagents and consumables is $6.14 for each test.

Title: Supplementary Data 3.
Description: CAD files for SPOT device assembly

A description of each component can be found in Figure 3.

Title: Supplementary Data 4.
Description: Sequences of oligos and DNA fragments
