# Supplementary material for: A rapid, accurate, scalable, and portable testing system for COVID-19 diagnosis
Source: Nat Commun. 2021 May 18;12:2905. doi: 10.1038/s41467-021-23185-x (PMC8131735; doi:10.1038/s41467-021-23185-x)
Supplement: Supplementary file 8 — Reporting Summary [file 41467_2021_23185_MOESM8_ESM.pdf]

nature research

Corresponding author(s): Huimin Zhao

Last updated by author(s): Apr 15, 2021

Reporting Summary

Nature Research wishes to improve the reproducibility of the work that we publish. This form provides structure for consistency and transparency in reporting. For further information on Nature Research policies, see our Editorial Policies and the Editorial Policy Checklist.

Statistics

For all statistical analyses, confirm that the following items are present in the figure legend, table legend, main text, or Methods section.

n/a	Confirmed	
☐	☒	The exact sample size (*n*) for each experimental group/condition, given as a discrete number and unit of measurement
☐	☒	A statement on whether measurements were taken from distinct samples or whether the same sample was measured repeatedly
☒	☐	The statistical test(s) used AND whether they are one- or two-sided<br>*Only common tests should be described solely by name; describe more complex techniques in the Methods section.*
☒	☐	A description of all covariates tested
☒	☐	A description of any assumptions or corrections, such as tests of normality and adjustment for multiple comparisons
☐	☒	A full description of the statistical parameters including central tendency (e.g. means) or other basic estimates (e.g. regression coefficient) AND variation (e.g. standard deviation) or associated estimates of uncertainty (e.g. confidence intervals)
☒	☐	For null hypothesis testing, the test statistic (e.g. *F*, *t*, *r*) with confidence intervals, effect sizes, degrees of freedom and *P* value noted<br>*Give P values as exact values whenever suitable.*
☒	☐	For Bayesian analysis, information on the choice of priors and Markov chain Monte Carlo settings
☒	☐	For hierarchical and complex designs, identification of the appropriate level for tests and full reporting of outcomes
☒	☐	Estimates of effect sizes (e.g. Cohen's *d*, Pearson's *r*), indicating how they were calculated

Our web collection on statistics for biologists contains articles on many of the points above.

Software and code

Policy information about availability of computer code

Data collection	SPOT portable testing device
Data analysis	Code of SPOT device microcontroller, display, fluorescent quantification, and data analysis (https://gitlab.engr.illinois.edu/ispot). Graphs were made by using the GraphPad Prism software (version 9.0.0).

For manuscripts utilizing custom algorithms or software that are central to the research but not yet described in published literature, software must be made available to editors and reviewers. We strongly encourage code deposition in a community repository (e.g. GitHub). See the Nature Research guidelines for submitting code & software for further information.

Data

Policy information about availability of data

All manuscripts must include a data availability statement. This statement should provide the following information, where applicable:

- Accession codes, unique identifiers, or web links for publicly available datasets
- A list of figures that have associated raw data
- A description of any restrictions on data availability

All data is available in the main text or the supplementary information text and files. Source data is provided with this paper. Fluorescent signal data from the SPOT device and qPCR machine are available from the corresponding author upon request, and can be found in Figshare (https://doi.org/10.6084/m9.figshare.14417639.v1) as well.

Field-specific reporting

Please select the one below that is the best fit for your research. If you are not sure, read the appropriate sections before making your selection.

☒ Life sciences ☐ Behavioural & social sciences ☐ Ecological, evolutionary & environmental sciences

For a reference copy of the document with all sections, see nature.com/documents/nr-reporting-summary-flat.pdf

Life sciences study design

All studies must disclose on these points even when the disclosure is negative.

Sample size	No statistical methods were used to predetermine sample size. To ensure experimental reliability and reproducibility, replicate runs (3 repeats or more) using IVT-RNA spiked saliva samples and gamma-irradiated virus spiked saliva samples for SPOT assay were conducted. For the reliability and reproducibility of LoD, 30 replicates were analyzed for each LoD we reported. For the testing of clinical samples, 104 samples were used in a blinded test. Our aim is just to demonstrate the feasibility of SPOT assay for SARS-CoV-2 diagnosis.
Data exclusions	No data was excluded.
Replication	Experiments were performed independently. At least three replicates were set up for each real-time and endpoint fluorescence detection of IVT-RNA templates and inactivated virus samples to demonstrate the findings were reliably reproduced. For the each LoD we reported, 30 more replicates were performed to ensure the reliability and reproducibility.
Randomization	We selected the target gene sites for SPOT detection assay according to the WHO and CDC recommendation. The target sequences are within both WHO (E gene) and CDC (N gene) assays targeted region. They have to be no-randomly selected in order to evaluate the detection performance of our established method.
Blinding	Validation on clinical samples is a blinded test. Clinical samples were tested by an individual who was blinded to the RT-PCR results.

Reporting for specific materials, systems and methods

We require information from authors about some types of materials, experimental systems and methods used in many studies. Here, indicate whether each material, system or method listed is relevant to your study. If you are not sure if a list item applies to your research, read the appropriate section before selecting a response.

Materials & experimental systems

n/a	Involved in the study	
☒	☐	Antibodies
☒	☐	Eukaryotic cell lines
☒	☐	Palaeontology and archaeology
☒	☐	Animals and other organisms
☐	☒	Human research participants
☒	☐	Clinical data
☒	☐	Dual use research of concern

Methods

n/a	Involved in the study	
☒	☐	ChIP-seq
☒	☐	Flow cytometry
☒	☐	MRI-based neuroimaging

Human research participants

Policy information about studies involving human research participants

Population characteristics	Provided 104 clinical human saliva samples were mixture of positive samples and negative samples, which were already analyzed by RT-PCR.
Recruitment	104 clinical human saliva samples were collected by Veterinary Diagnostic Laboratory of University of Illinois at Urbana-Champaign from the community of Urbana-Champaign. They have obtained informed consent from all participants.
Ethics oversight	Clinical trial study was registered as an IBC project with the project number IBC-4609 in the Division of Research Safety, University of Illinois at Urbana-Champaign.

Note that full information on the approval of the study protocol must also be provided in the manuscript.
